# MECHANISMS IN ENDOCRINOLOGY: The gut–brain axis: regulating energy balance independent of food intake

**DOI:** 10.1530/EJE-21-0277

**Published:** 2021-07-14

**Authors:** Ruben Nogueiras

**Affiliations:** 1 Department of Physiology, CIMUS, USC, CIBER Fisiopatología Obesidad y Nutrición (CiberOBN), Instituto Salud Carlos III, Galician Agency of Innovation, Xunta de Galicia, Santiago de Compostela, Spain

## Abstract

Obesity is a global pandemic with a large health and economic burden worldwide. Bodyweight is regulated by the ability of the CNS, and especially the hypothalamus, to orchestrate the function of peripheral organs that play a key role in metabolism. Gut hormones play a fundamental role in the regulation of energy balance, as they modulate not only feeding behavior but also energy expenditure and nutrient partitioning. This review examines the recent discoveries about hormones produced in the stomach and gut, which have been reported to regulate food intake and energy expenditure in preclinical models. Some of these hormones act on the hypothalamus to modulate thermogenesis and adiposity in a food intake-independent fashion. Finally, the association of these gut hormones to eating, energy expenditure, and weight loss after bariatric surgery in humans is discussed.

## Invited Author’s profile


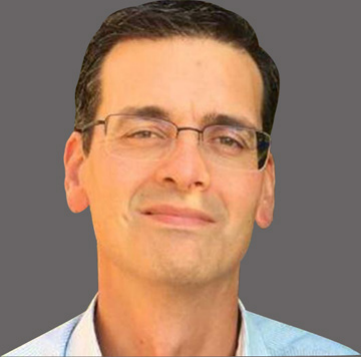


**Ruben Nogueiras** is currently an Oportunius Research Professor and Associate Professor of the University of Santiago de Compostela (Spain). He coordinates the Molecular Metabolism Group in the CIMUS. The group is focused on the study of molecular mechanisms involved in obesity and its associated diseases such as type 2 diabetes and metabolic-associated fatty liver disease (MAFLD). More particularly, our two lines of research aim to increase our understanding of the crosstalk between the hypothalamus and peripheral organs and to find new molecules involved in both glucose and lipid metabolism in the liver. The translational value of our basic science research data can be determined by combining our preclinical results with clinical data.

## Introduction

Obesity has already reached pandemic proportions worldwide, yet the percentage of people defined as obese appears to be still increasing (1, 2, 3, 4, 5). According to the World Health Organization, worldwide obesity has nearly tripled since 1975; in 2016, more than 1.9 billion adults were overweight, and more than 650 million, obese. In keeping with these numbers, 39% of all adults were overweight, and 13% were obese, worldwide. This rapid increase in obesity is likely due to multiple factors, such as social and environmental determinants and genetic predisposition. In the simplest terms, however, the underlying basis of obesity is a higher energy intake than energy expenditure (6, 7). In addition, obesity is commonly associated with several diseases, such as type-2 diabetes, fatty liver (1, 2, 4, 8, 9, 10), and different types of cancer (11), which overall constitute metabolic syndrome.

The CNS modulates energy balance by acting through both the brain and peripheral organs (12, 13, 14, 15); this was demonstrated several decades ago, based on the observation that animals with hypothalamic lesions have increased adiposity. In terms of energy homeostasis regulation, the hypothalamus is the most studied area of the CNS. The hypothalamus modulates the levels of the neurotransmitters and neuromodulators that control food intake and energy expenditure in response to changes in energy status (16, 17, 18, 19, 20). These peripheral metabolic signals (such as metabolites, nutrients, and hormones) reach hypothalamic neurons, where they regulate neuronal activity and/or the expression and synthesis of neuropeptides and neurotransmitters (16, 17, 18, 19, 20).

We now understand that the gut not only functions in digestion and assimilation of nutrients but also contains endocrine tissues that are involved in the regulation of energy balance. This gut regulation is carried out by different hormones secreted from endocrine cells in the gastrointestinal tract, as well as by several neural pathways that communicate information from the signals responsible for the regulation of food intake and energy expenditure. After receiving the information from peripheral tissues, the CNS processes these signals and sends orders to the controllers of energy homeostasis. Notably, hormones produced in the gastrointestinal tract include peptides secreted not only by the gut but also by the pancreas and liver. This review will focus on the effects that some of the peptide hormones (mainly those secreted by the stomach and gut) exert in energy homeostasis, which regulate body weight by mechanisms that affect not only food intake but also energy homeostasis and fat storage, among others.

## Hypothalamus and energy balance

The hypothalamus lies just beneath the thalamus and above the pituitary gland, to which it is attached by a stalk. It modulates a broad spectrum of metabolic functions, including the function of endocrine axes and energy homeostasis. Structurally, the hypothalamus comprises several nuclei, which constitute interconnected neuronal circuits via axonal projections (16, 17, 18, 19, 20). The importance of the hypothalamus in the regulation of body weight was initially reported by lesion studies, which showed that damaging the ventromedial hypothalamus (VMH) causes hyperphagia and obesity while damaging the lateral hypothalamus (LH) leads to aphagia and even death by starvation.

The hypothalamus also contains other nuclei, including the arcuate nucleus (ARC), the dorsomedial nucleus (DMH), and the paraventricular nucleus (PVH), which also participate in the control of energy homeostasis. The ARC is a central region in controlling both food intake and energy expenditure. It integrates signals from the periphery based on its unique anatomical position and the relatively high permeability of the blood–brain barrier in this area. Here, different factors from the periphery are sensed by two main neuronal populations in the ARC: (i) neurons that express neuropeptides stimulate appetite, such as agouti-related protein (AgRP) and neuropeptide Y (NPY) and ii) neurons that express neuropeptides inhibit feeding, including proopiomelanocortin (POMC), the precursor of alpha-melanocyte-stimulating hormone (α-MSH), and the cocaine- and amphetamine-regulated transcript (CART) (21). Finally, the VMH is one of the hypothalamic areas that is most involved in the thermogenic activity of brown adipose tissue (BAT), involved in the sympathetic nervous system (SNS) outflow to BAT (22).

## White, brown, and beige adipose tissues

White adipose tissue (WAT) comprises white adipocytes and the stromal vascular fraction of cells (preadipocytes, fibroblasts, endothelial cells, etc). While its main role is to store energy in the form of triglycerides, it is also an important endocrine organ. In contrast, BAT is responsible for energy and dissipation and is the most important organ for non-shivering thermogenesis. Brown adipocytes commonly contain multilocular lipid droplets and numerous, enlarged mitochondria and are abundantly innervated by sympathetic nerve efferent fibers that allow them to regulate thermogenesis. The physiological relevance of BAT in humans has only recently been elucidated. BAT is especially abundant in newborns and in hibernating mammals, so it was classically assumed that this tissue only plays an important thermogenic function in these situations (23, 24, 25, 26). However, metabolically active BAT is also present in cervical, supraclavicular, and paravertebral regions in healthy adults (24, 27, 28, 29, 30). A third class of adipose tissue, named beige/brite adipose tissue, has now also been identified (31, 32, 33). Beige adipocytes have similar morphologic features to brown adipocytes (e.g. have central nuclei, multilocular lipid droplets, and numerous mitochondria and are responsive to thermogenic stimuli). WAT can convert (to a degree) into brown adipocyte-like cells (beige, or similar) upon sustained cold exposure or direct β-adrenergic activation, in a process termed 'browning'. However, in contrast to brown adipocytes, beige adipocytes are located within the WAT depots. It is now accepted that both BAT and brite/beige adipocytes coexist in adults (34, 35).

## Ghrelin

Ghrelin is a 28-amino acid (aa) peptide originally discovered as the endogenous ligand for the 'orphan' growth hormone secretagogue receptor (GHSR) (36). Ghrelin is produced mainly by the stomach (36) but also by other tissues, such as the duodenum (37); its production is regulated by nutritional factors as well as different hormone factors. Fasting leads to increased ghrelin expression in the stomach as well as increased ghrelin plasma concentrations (38); its levels are reduced immediately after food intake. Postprandial ghrelin reduction was initially reported to be proportional to the ingested calorie load (39). Although the postprandial ghrelin response to macronutrient composition in people with obesity is not very clear (40, 41, 42), it is macronutrient-specific in people in the normal-weight range, with isoenergetic meals of different macronutrient content differentially affecting ghrelin levels. In general, carbohydrate intake leads to the most rapid ghrelin reduction; protein intake induces prolonged ghrelin suppression, and fat intake only minimally affects ghrelin levels (41, 43). Ghrelin levels change throughout the day, reaching high levels before food intake and during the night, suggesting that ghrelin is an important factor in meal initiation (44). Circulating ghrelin levels are decreased in human and rodent obesity (45, 46, 47) and are known to be elevated in people with anorexia nervosa and in states of cachexia (48, 49). Ghrelin is the only known peptide hormone secreted by the gastrointestinal tract that induces food intake and adiposity (50). Evaluation of the relationship between ghrelin and energy expenditure in humans shows an inverse relationship between ghrelin and the resting metabolic rate in lean, obese, and hyperthyroid subjects (51, 52). In addition, there is also an inverse relationship between ghrelin in the resting and postprandial energy expenditure, which (in healthy, young women) seems to be independent of variations in body composition, insulin levels, and daily energy intake (53). This relationship between active ghrelin levels and energy expenditure appears to be important in human obesity (51). Bariatric surgery, currently the most efficient treatment for obesity, enhances glucose homeostasis and enhances gut hormone secretion immediately after surgery, but its effects on the serum ghrelin levels following bariatric surgery remain controversial. Different studies have reported serum ghrelin levels to be increased, decreases, or not changed, following bariatric surgery (for specific reviews on this topic, see (54, 55, 56)). Overall, a comparison between the studies is difficult, given the different anthropometric characteristics of the patients, the distinct types of surgery, and the different ghrelin assays used. Therefore, it remains unclear whether changes in ghrelin levels have any impact on weight loss after bariatric surgery (Table 1).

**Table 1:** Structure, site of production, main functions, and levels following bariatric surgery of different gut hormones.

Hormone	Structure	Site of production	Main functions	Levels post bariatric surgery
Ghrelin	28 aa	Stomach	Stimulates food intake and adiposity, increases gastric emptying	Controversial
GLP1	31 aa	Intestinal L cells	Stimulates insulin, inhibits food intake, reduces gastric emptying	↑
GLP2	33 aa	Intestinal L cells	In humans, no effect on food intake or gastric emptying	↑
Oxyntomodulin	37 aa	Intestinal L cells	Inhibits food intake, reduces gastric emptying	↑
GIP	42 aa	Intestinal K cells	Stimulates insulin, inhibits food intake	↑
PYY	36 aa	L cells in the ileum and colon	Inhibits food intake, reduces gastric emptying	↑
CCK	115 aa	L cells in colon	Inhibits food intake, slows gastric emptying, pancreatic enzyme secretion	↑
Uroguanylin	16 aa	Intestinal epithelial cells Increases secretion of Na, Cl, and HCO3	Inhibits food intake, increases gastric motility	↑
FGF15/19	216 aa	Ileum	Inhibits food intake, increases gastric motility	↑

Even though the potent anabolic action of ghrelin is very clear, preclinical studies show that manipulating the ghrelin/GHSR system in genetically modified mice has only a mild phenotype. Specifically, mice with a genetic knockdown (KO) of the ghrelin gene (Ghrl^−/−^) do not differ in food intake, body weight, body size, growth rate, body composition, reproduction, bone density, or organ weights, as compared to WT mice (57, 58). However, chronically exposing the Ghrl^−/−^ mice (with a mixed genetic background) to a high-fat diet (HFD) shows a clear metabolic benefit from being ghrelin-deficient, especially if the HFD starts at an early age (note that this benefit is not evident in mature, congenic KO mice with a pure C57BL/6J background (59)). Namely, Ghrl^−/−^ mice (with a genetically mixed background) fed a HFD have a reduced respiratory quotient and increased fat oxidation, indicating a shift in the metabolic fuel preference toward higher lipid utilization (58). Further, these Ghrl^−/−^ mice have lower body weights and less fat mass despite similar food intake as the WT mice, which might be attributed to increased energy expenditure and locomotor activity (60). Overall, these results indicate that ghrelin can play a role in the preference of nutrients using metabolic fuels other than fat.

*Ghsr1a*^−/−^ mice exhibit similar growth curves and food intake as their WT littermates (61) but have a modest reduction of bodyweight in adulthood (despite similar food intake, bone density, body composition, and metabolic rates) and lower levels of insulin growth factor-1 (IGF1) (61). Interestingly, *Ghsr1a*^−/−^ mice are not able to maintain glucose levels to the same extent as WT animals when they are fasted or challenged by calorie restriction, indicating that GHSR1A is essential for maintaining glucose metabolism under conditions of negative energy balance (59). The age of the animals seems to be a crucial factor controlling the endogenous role of GHSR1a. For instance, 4-month-old *Ghsr1a*^−/−^ mice show a slight reduction of body weight, which increases with age (to a modest reduction) (62). Age-induced obesity is mainly produced by an increase of adipogenesis in WAT and a decline in the thermogenic capacity of BAT. Surprisingly, however, older *Ghsr1a*^−/−^ mice exhibit a lean phenotype with increased insulin sensitivity, reduced fat mass, and a healthier lipid profile (62). The reason for reduced adiposity is that old *Ghsr1a*^−/−^ mice exhibit elevated energy expenditure, increased resting metabolic rate, and increased expression of thermogenic genes in BAT (62). Notably, double-KO mice lacking both ghrelin and GHSR1a have metabolic improvements, defined as lower body weight, reduced body fat, and lower plasma cholesterol, than WT mice when fed a standard chow diet (63). In other words, the lack of both ghrelin and GHSR1a increases energy expenditure, body core temperature, and locomotor activity despite normal food intake (63).

**Figure 1:**
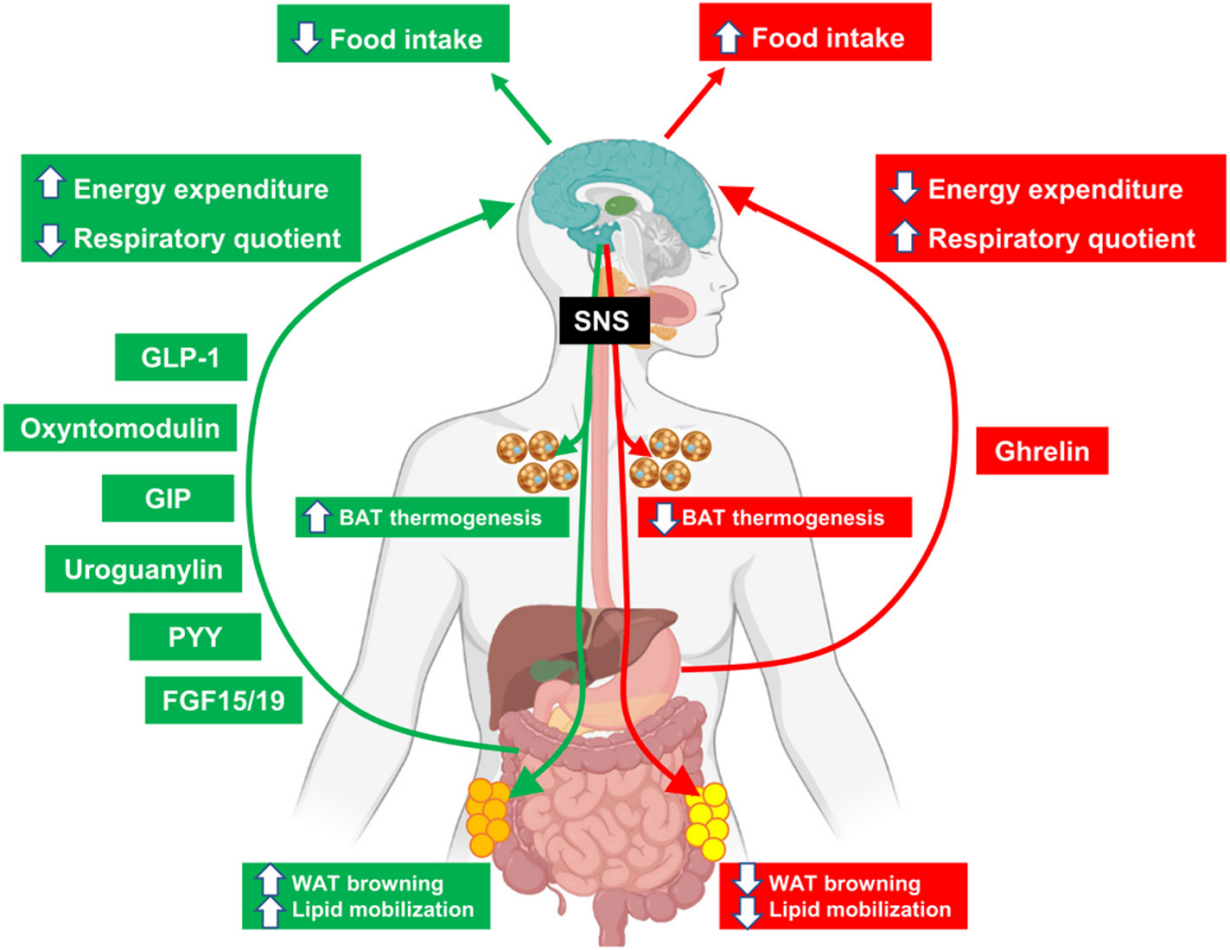
Schematic representation of the metabolic effects of (i) catabolic hormones, including glucagon-like peptide-1 (GLP1), oxyntomodulin, gastric inhibitory polypeptide (GIP), and uroguanylin), and (ii) anabolic hormones, such as ghrelin. These hormones act at the central level: receptors of each hormone are expressed in different brain areas (of which the hypothalamus is the most studied) and can directly modify the metabolism of different organs via the sympathetic nervous system (SNS).

It is worth mentioning again that ghrelin is the only circulating gut hormone that, upon systemic and central administration, potently increases not only adiposity and food intake (50) but also other parameters, such as energy expenditure and nutrient partitioning (64, 65), that are relevant for its anabolic effect (Fig. 1). To exert these broad biological actions, ghrelin requires neuronal circuits located in different brain areas, and especially in the hypothalamus (66). Ghrelin increases the activity of *Npy-* and *Agrp-*expressing neurons and inhibits the activities of *Pomc*-expressing neurons (64). NPY and AgRP are crucial for ghrelin’s effects (67). Thus, ghrelin’s effects on adiposity are achieved through both centrally and peripherally mediated signaling mechanisms that modulate (i) the hypothalamic melanocortin system, and (ii) peripheral lipid metabolism, in a manner that is independent of both food intake and growth hormone (68, 69, 70). Central administration of ghrelin induces increased adiposity by stimulating key enzymes that promote fatty acid storage as well as by decreasing the expression of genes that control the rate-limiting step in fat oxidation (69, 71). The actions of the brain ghrelin system on adipose tissue are mediated by the SNS and are independent of food intake or energy expenditure (69).

## Proglucagon-derived hormones

The proglucagon gene is expressed by the pancreatic islet α-cells, which are specific enteroendocrine cells (L-cells) of the intestinal mucosa, and by a discrete set of neurons within the nucleus of the solitary tract. The proglucagon gene encodes structurally related proglucagon-derived peptides, including glucagon, glucagon-like peptide-1 (GLP1), glucagon-like peptide-2 (GLP2), glicentin, and oxyntomodulin (Oxm). The relative amounts and forms of these peptides in different cell types depend on tissue-specific posttranslational modifications by prohormone convertases. For instance, in pancreatic α-cells, prohormone convertase 2 produces predominantly glucagon, while in the intestinal L-cells and neurons of the nucleus of the solitary tract, prohormone convertases 1/3 produce GLP1, oxyntomodulin, and GLP2 (72, 73, 74, 75) (for a detailed review on this topic, see (76)).

## GLP1

GLP1 is a 31-aa long (7-37) peptide hormone produced and secreted by intestinal enteroendocrine L-cells and certain neurons within the nucleus of the solitary tract in the brainstem. Further processing by a prohormone convertase results in peptides of 36 and 30 amino acids, GLP1(1-36)amide and GLP1(7-36)amide (77). GLP1 regulates blood glucose levels through its combined actions on the stimulation of glucose-dependent insulin secretion and the inhibition of glucagon secretion, gastric emptying, and food intake (78). The major stimulus for GLP1 secretion is the ingestion of nutrients, including glucose and fatty acids. GLP1 also inhibits food intake and promotes satiety in normal, obese, and diabetic humans (79). In addition to its clear effects on feeding, some reports have shown that GLP1R analogs (like liraglutide) can also increase energy expenditure (80, 81, 82, 83, 84); in contrast, however, another study showed that 12-week treatment with liraglutide can reduce energy expenditure, with a tendency to be decreased that persisted after 26 weeks, without affecting the fat fraction in the supraclavicular BAT depot (85). While still controversial, these findings suggest that liraglutide-induced weight loss is based on a reduction in energy intake rather than an increase in energy expenditure. Concerning bariatric surgery, different studies have shown that GLP1 plays an important role in the weight loss-independent glycemic effects of bariatric surgery (in particular, for Roux-en-Y gastric bypass and sleeve gastrectomy). In general, there is a marked postprandial rise in GLP1 levels after bariatric surgery, both in animal models and humans. This is a consequence of rapid nutrient delivery in the gastrointestinal tract, where the majority of L-cells are located. However, it is not clear that the high postprandial GLP1 after surgery is responsible for postoperative metabolic improvements (for a review on this topic, see (86, 87)) (Table 1).

In rodents, central or peripheral administration of GLP1R agonists inhibits food intake and leads to a reduction in body weight (88, 89). There are abundant reports showing that GLP1R analogs stimulate energy expenditure in a feeding-independent manner. For instance, the direct stimulation of the brain GLP1 signaling pathway by GLP1 or liraglutide reduces body weight and increases the thermogenic activity of BAT and the browning of WAT in a food intake-independent manner (82, 90). Indeed, this stimulation of the thermogenic activity was also reflected by the augmented levels of peroxisome proliferator-activated receptor-gamma coactivator-1alpha (PGC1A) and uncoupling protein-1 (UCP1), two surrogate markers of thermogenesis, in the BAT of animals receiving GLP1 receptor agonists (90). This response was abrogated in GLP1 receptor null mice, indicating the specificity of the GLP1/GLP1 receptor system in the regulation of the thermogenic function. The stimulatory effect of brain GLP-1 on BAT activity is mediated by the SNS, and the brain administration of GLP1 receptor agonists enhances the electrophysiological activity of the sympathetic fibers that innervate the interscapular BAT (90) (Fig. 1).

GLP1R is widely expressed in different neuronal subsets within the CNS, coexisting with neuronal populations that play a key role in regulating energy homeostasis (91, 92). Injection of liraglutide into the ventromedial nucleus (VMH) recapitulates the effects found after ICV injection of liraglutide as well as the thermogenic program in WAT (82). On the other hand, when GLP1 is administered into the CNS over several days, it also decreases lipid deposition in adipocytes of lean mice. These actions seem to be partially modulated through effects on the sympathetic outflow, leading to an efficient shift in substrate metabolism and reduced energy storage and adiposity (93). However, in conditions of obesity, there seems to be a selective central GLP1–resistance; even though the brain GLP1 system is efficiently regulating food intake, body weight, and glucose homeostasis (94), it has no capacity to exert its actions on adipocyte metabolism. Indeed, the potential clinical relevance of the molecular mechanism described in rodents remains unknown, and the importance of the brain GLP1R signaling for the weight loss and antidiabetic effects of GLP1 receptor agonists remains to be established.

## GLP2

GLP2 is a 33-aa peptide released from the mucosal enteroendocrine L-cells of the intestine by cleavage with the proglucagon prohormone convertase 1/3. It is secreted following nutrient ingestion, and reduces gastric emptying, enhances digestion and absorption, and is involved in the maintaining of glucose homeostasis (95, 96). Peripheral administration of GLP2 reduces short-term food intake in mice (97), and this action may be mediated by both peripheral and central mechanisms. On one hand, the action of GLP2 on gastric distension inhibits feeding via vagal afferent neurons; indeed, the GLP2 receptor is found in the cell bodies of vagal afferents in the nodose ganglion (98). On the other hand, the GLP2 receptor is also located in POMC neurons, where its activation suppresses feeding (99) and also enhances hepatic insulin sensitivity (95). This anorexigenic effect of GLP2 does not occur in diet-induced obese mice, indicating a GLP2 resistance in obesity (97).

Even though the effects of GLP2 are clear in rodents, peripheral administration of GLP2 in humans has no effect on gastric emptying, satiety, or energy intake (100, 101). However, in humans, GLP2 can inhibit both pentagastrin-stimulated and sham feeding-stimulated gastric acid secretion (102, 103), and it can also stimulate glucagon secretion and enhance lipid absorption (103). Similar to GLP1, GLP2 levels rise and are correlated with satiety after bariatric surgery (104, 105, 106). However, another study in obese adolescents failed to show any effects of bariatric surgery son GLP2 (107) (Table 1).

## Oxyntomodulin (Oxm)

Oxm is a 37-aa peptide hormone found in the colon that is produced by the oxyntic cells of the oxyntic mucosa. Similar to GLP1, Oxm is rapidly released after food ingestion from the L-cells of the distal small intestine, in proportion to meal calorie intake (72, 73, 74). Oxm is reported to be a full agonist of GLP1R and the glucagon receptor, although it has a reduced affinity as compared to GLP1 or glucagon (108, 109, 110, 111, 112). Oxm causes weight loss in humans (113, 114) and rodents (110, 115). Overweight and obese people who receive s.c. administration over a 4-week period showed an average weight loss of 2.3 kg (114). A randomized controlled trial showed that the s.c. administration of pre-prandial oxyntomodulin increases energy expenditure, and reduces energy intake, in overweight and obese humans (116). Oxm levels remained unchanged between a group of obese women after bariatric surgery or women losing weight by diet, but its levels were elevated in response to oral glucose after surgery but not after diet (117). In addition, an increase in postprandial levels of Oxm following bariatric surgery has been suggested as a predictor of weight loss after surgery (118, 119) (Table 1).

In mice, Oxm lowers food intake, reduces body weight, and increases core temperature as compared to control animals that received equivalent amounts of food; this weight loss is associated with increased energy expenditure (110, 115). Therefore, the bodyweight-lowering effects of Oxm are likely involved in suppressed food intake and increased energy expenditure (Fig. 1).

## Gastric inhibitory polypeptide (GIP)

GIP is a 42-aa peptide produced in, and secreted from, intestinal K cells of the proximal small intestine (duodenum and jejunum) in response to eating (120). The GIP receptor is abundantly expressed in pancreatic β cells, where its activation provokes an insulin response (121). In humans, an infusion of GIP does not cause any significant effect on appetite, energy intake, or energy expenditure (122, 123). Likewise, based on rodent preclinical work (see below), studies investigating the effects of a co-infusion of GIP and GLP1 in humans found that GIP does not potentiate the effects of GLP1 on lowering energy intake (123, 124) (Table 1).

In rodents, chronic exposure to a high-fat diet (HFD) increases intestinal expression of GIP (125), induces K cell hyperplasia (126), and increases the presence of GIP in circulation (126). Further, human obesity correlates with hypersecretion of GIP (127). The physiological role of GIP in metabolic control beyond that of an endogenous incretin is controversial. Studies report opposite effects on the maintenance of body weight in various loss- or gain-of-function models (see review (128)). The use of genetic animal models has suggested that GIP promotes obesity. For instance, Gipr^–/–^ mice are protected from obesity (either diet-induced or leptin deficiency-induced), maintain proper insulin sensitivity, and use more fat as an energy substrate than WT controls, despite exhibiting no differences in food intake (129). Other studies suggest a role for GIPR agonism in promoting fat storage. GIP induces the expression of the lipogenic machinery (130), stimulates the adipose tissue lipoprotein lipase activity (131), and enhances the uptake of glucose and free fatty acids (132). In addition, knock-in of a biologically inactive GIP impairs oral glucose tolerance and protects from diet-induced obesity and insulin resistance in mice (133). Similarly, mice lacking GIP-producing cells present attenuated weight gain with a HFD and consume more energy than WT controls, recapitulating many of the phenotypic observations of Gipr^–/–^ mice (134). In contrast to studies indicating that GIP favors anabolic action, several recent studies have suggested that GIP exerts a beneficial effect on adipose tissue and promotes weight loss. For instance, transgenic *Gip* overexpression protects mice against diet-induced obesity; their weight loss is caused by reduced food intake while energy expenditure remains unaffected (135). This was associated to an upregulation of the *Gip* gene expression in the VMH (135). The relevance of the hypothalamic neuronal circuits in mediating the effects of GIP was corroborated in a pharmacological report, in which GIP was administered directly to the brain; in this case, there was no association to differences in food intake, yet the GIP-treated mice showed a decreased body weight as compared to controls (136). These findings thereby suggest that GIP stimulates energy expenditure (Fig. 1). Notably, the feeding intake differs depending on whether if GIP is administrated to the brain or is specifically injected in the VMH, suggesting that there are hypothalamic nuclei-specific mechanisms that regulate specific effects of GIP in energy balance.

## Peptide YY_3-36_ (PYY)

PYY, also known as peptide tyrosine tyrosine, is a 36-aa peptide released from cells in the ileum and colon. PYY is part of a larger family of proteins that also includes NPY and pancreatic polypeptide. All three peptides contain a hairpin turn within the amino acid backbone and hence are known as pancreatic polypeptide-fold proteins. PYY_3–36_ is a major form of PYY in both the gut mucosal endocrine cells and in circulation (137). PYY is secreted in response to feeding in proportion to meal energy content (138). Peripheral administration of PYY_3-36_ inhibits food intake for several hours in both rodents and humans (139, 140, 141). It also causes weight loss in obese models, such as leptin-deficient mice, diet-induced obese mice, and non-diabetic fatty Zucker rats (139, 142). The inhibitory effect of PYY on feeding involves mechanisms regulating appetite within the CNS, as PYY_3-36_ modulates brain activity in appetite centers in humans (143). Interestingly, the co-infusion of PYY_3-36_ and GLP1 has a synergistic action in reducing energy intake in overweight men, as this reduction was higher than the injection of each peptide separately (144).

In addition to its relevant action in feeding, PYY_3–36_ has also been reported to affect energy expenditure, but the different studies show controversial data. For instance, infusions of PYY_3–36_ were shown to increase energy expenditure and fat oxidation in both obese and lean humans (145), but this effect was not reproduced in another study in obese humans, as s.c. infusion of PYY did not affect resting energy expenditure (146). Also, another report failed to detect changes in energy expenditure after the administration of PYY_3–36_ in overweight men (144). In healthy women, total PYY was significantly correlated to postprandial energy expenditure at 60, 90, 120, and 150 min post-treatment (147); these results are supported by an independent work in normal-weight premenopausal women, which showed a significant association between PYY and the resting metabolic rate (148), which is an important contributor to energy expenditure. In summary, although some studies suggest a potential role for PYY in the regulation of energy expenditure in humans, it seems that this action may be dependent on the characteristics of the cohort; clarifying this deserves further research. On the other hand, PYY levels have been reported to increase following bariatric surgery. Both fasting and postprandial PYY levels increase significantly within just 1 week of surgery in obese patients and remain elevated after 1 year (149, 150, 151, 152) (Table 1).

## Cholecystokinin (CCK)

CCK is synthesized and secreted by enteroendocrine cells in the duodenum mainly in the presence of digestion products of fats and proteins. Human CCK preproprotein contains 115 aa, but posttranslational processing can result in the different molecular forms identified in tissue and blood, which range in size from 4 to 83 aa. CCK8 is the most abundant form of CCK in the human brain, while CCK8, CCK22, CCK33, and CCK58 are present in the human intestine and circulation in significant amounts (whereby CCK22 and CCK33 are the most abundant in circulation) (153). The presence of CCK causes the release of digestive enzymes and bile from the pancreas and gallbladder, respectively and also acts as a hunger suppressant (154). Administration of CCK inhibits food intake in humans as well other animals. CC suppresses energy intake in a dose-dependent manner in rats (155), and CCK8 reduces both the size and duration of a meal (156). In humans, i.v. infusion of CCK8 and CCK33 increases the perception of fullness, decreases hunger, and reduces energy intake (157, 158, 159, 160). However, the response of CCK in humans following bariatric surgery has been reported differently according to the studies; while one report found that CCK levels are not altered after a glucose or protein meal after surgery (161), another one that compared patients before and after bariatric surgery with healthy lean volunteer controls found that post-surgery patients have higher levels of postprandial CCK (162) (Table 1).

Although there is no clear evidence for the role of CCK in the modulation of energy expenditure in humans, animal models suggest an inhibitory effect of CCK on this parameter. CCK- and CCK2R-deficient mice display increased energy expenditure (163, 164). CCK2R knockout mice have a higher energy expenditure, which seems to be linked to increased physical activity (164); in contrast, CCK-deficient mice are resistant to HFD-induced obesity by mechanisms independent of energy intake and physical activity, which remained unchanged (163). This resistance against diet-induced obesity is also associated to defects in fat absorption, especially of long-chain saturated fatty acids (163). Further work is necessary to clarify the physiological mechanisms and nutrient conditions by which CCK may affect energy expenditure in rodents and to address whether these preclinical data may have some clinical implications for humans.

## Uroguanylin

Uroguanylin (UGN) is a 16-aa peptide encoded by the precursor pro-UGN, which is secreted by duodenal epithelial cells into the lumen, where it is processed and converted into its active form. UGN binds to the transmembrane receptor guanylyl cyclase 2C (GUCY2C), triggering intracellular levels of cyclic GMP (cGMP) (165, 166). Guanylin is a 15-aa peptide also derived from the prohormone pro-UGN, secreted by epithelial cells from the colon, that binds to GUCY2C (167, 168). The activation of GUCY2C increases intestinal secretions of the electrolytes sodium, chloride, and bicarbonate (169, 170). Excessive synthesis of UGN or guanylin causes diarrhea; indeed, staphylococcal enterotoxins (STs) are bacterial proteins linked to significant human diseases (e.g. food poisoning and toxic shock syndrome) that bind to and functionally activate the GUCY2C receptor (167, 171).

UGN expression in the intestine is modulated by the availability of nutrients and correlates positively with circulating UGN levels (172). In mice, circulating levels of pro-UGN are higher in response to the ingestion of nutrients (165). In agreement with a postprandial release of UGN, nutrient deprivation of mice reduces the expression of UGN in the duodenum as well as the circulating levels of UGN (173). According to this, the refeeding of pre-fasted mice returns the levels of UGN in the intestine and blood to baseline conditions. The nutrient-dependent adjustment of UGN levels is mediated by leptin, an adipose tissue-derived hormone whose expression is positively correlated with the volume of fat mass. In mice, leptin levels decreased in fasting states and are restored after re-feeding (173).

UGN has been also suggested to reduce food intake and body weight in rodents, after circulating UGN reaches the hypothalamus and binds to the GUCY2C receptor (165). The systemic and central injection of UGN or compounds activating the GUCY2C receptor inhibits food intake in WT mice but have no effect in mice lacking GUCY2C (165). Despite these initial findings, results from subsequent studies have been controversial. While the initial report showed that GUCY2C receptor (Gucy2c–/–) deficient mice have increased adiposity due to hyperphagia (165), a later report was not able to reproduce those results and found that Gucy2c–/– mice did not differ from control mice in their body weight, fat mass, or glucose homeostasis (174). Thus, the endogenous relevance of the UGN–GUCY2C system for regulating energy balance has yet to be determined. The chronic central infusion of UGN in diet-induced obese mice over several days causes a food intake-independent reduction in weight gain and adiposity (175). The resistance against HFD-induced obesity could be explained by a higher energy expenditure, which is consistent with the stimulated activity of the BAT, as shown by augmented expression of genes necessary for thermogenesis (uncoupling protein 1, uncoupling protein 3, PR/SET domain 16, and peroxisome proliferator-activated receptor-gamma coactivator 1-alpha) in the BAT of UGN-treated obese mice (175). Furthermore, central administration of UGN not only induces browning but also stimulates lipid mobilization in s.c. adipose tissue, as shown by the increased levels of phosphorylated hormone-sensitive lipase (pHSL) (175) (Fig. 1). To exert its actions in WAT and BAT, UGN requires an intact SNS, as the lack or pharmacological blockade of beta-adrenoreceptors abolishes the catabolic effect of brain UGN in diet-induced obese mice. However, the effects of centrally administered UGN to mice are not only mediated by the sympathetic activity of fibers innervating adipose tissue but also via gastrointestinal effects. For instance, UGN stimulates gastric motility. The gastrointestinal action of UGN is mediated by the parasympathetic nervous system, and more precisely, by the vagus nerve that connects the gut with brain areas (176). Indeed, the UGN-induced gastric motility is blunted in vagotomized mice; however, in these mice, the excitatory action of UGN on the sympathetic nervous system persists, and therefore, they still show increased BAT thermogenic activity and browning of WAT (175). Overall, these results suggest that the chronic effects of UGN on metabolism occur via both branches of the autonomic nervous system.

## Fibroblast growth factor 15/19 (FGF15/19)

FGF15 in mice (or its human orthologue FGF19) is a 216-aa peptide hormone secreted in the absorptive cells of the mouse ileum that plays an important role in feedback inhibition of hepatic bile acid synthesis (177, 178). It is released from the gut after food intake and controls the homeostasis of bile acids and glucose during the transition from a fed to a fasted state (178, 179). Different reports have described that FGF15 acts in the brain of rodent models of obesity and enhances insulin sensitivity, improves glucose tolerance, decreases food intake and body weight, and increases energy expenditure (180, 181, 182, 183). Similar to other hormones produced in the gut, FGF15/19 levels are elevated after bariatric surgical procedures (184, 185, 186, 187, 188, 189, 190), and whilst most reports suggest an important role of this hormone in the metabolic benefits of bariatric surgery, one did not, as the increase of FGF19 did not parallel the improvement of glucose tolerance (189).

Although the pharmacological effects of FGF19 are well recognized in preclinical models, the physiological roles of FGF19 in humans remain to be clarified. In people with impaired fasting glucose, fasting FGF19 levels are decreased and are negatively correlated with fasting plasma glucose, but not associated with insulin secretion and sensitivity (191). A subsequent study described that FGF19 positively correlates with glucose effectiveness and is negatively associated with fasting plasma glucose and hepatic glucose production (192). FGF19 levels have been also described to be higher after bariatric surgery. Vertical sleeve gastrectomy, duodenal-jejunal bypass liner, and Roux-en-Y gastric bypass, but not gastric banding, significantly raises blood FGF19 levels, with an inverse association between FGF19 and BMI reduction post-surgery (193). Whether FGF19 can modify eating patterns in humans, and whether it is linked to energy expenditure, have not yet been assessed (Table 1).

## Concluding remarks

We now know that the gut acts as a nutrient sensor that can signal the release of several hormones. These signals about hunger or satiety produced long-term and/or short-term responses to control feeding behavior, acting through different peripheral and central mechanisms. Thus, energy intake, energy expenditure, and (as a result) body weight are tightly regulated. So far, several lifestyle interventions, bariatric surgery, and some drugs are currently used to treat obesity, but all have associated problems, and most – if not all – pharmacological agents have limited effectiveness.

The continued research on the broad spectrum of mechanisms regulating energy balance, including the gut–brain axis, underscores the difficulties faced by an obese person who is trying to lose weight. For instance, because ghrelin stimulates appetite in rodents and humans, it was expected that ghrelin antagonists could exert beneficial effects against obesity; however, this strategy has failed (194, 195). Also, different approaches with drugs that individually activate the receptors for different catabolic gut hormones have not provided the expected beneficial results, and the GLP1 analog liraglutide has been the only one to be approved by the US Food and Drug Administration (FDA) and the European Medicines Evaluation Agency (EMEA) for the treatment of obesity. In the past few years, much effort has been put into developing combinatorial approaches that target food intake and energy expenditure pathways (including those directed toward insulin sensitivity), which have shown very promising results (196, 197). Some of these approaches combine gut peptide agonism to treat obesity and its associated disorders and have been demonstrated to be effective at reducing weight, improving glucose homeostasis, and ameliorating the content of fatty acids in the liver of diet-induced obese mice (198, 199, 200). Some of the gut peptide-based multi-agonists initially investigated in preclinical models of obesity are currently under phase I or II clinical trials (see review (196)), which will likely produce the main next-generation pharmacological agents to be used. These new drugs include semaglutide, a second-generation GLP1 agonist, and tirzepatide, a dual agonist for GLP1 receptor and GIP receptor (201, 202); both of these seem to improve the weight loss effect as compared to the currently approved drugs, which produce an average weight loss of 5% to 7%. Whether these drugs act on the brain of people with obesity has not been assessed; however, given that receptors for most (if not all) gastrointestinal hormones are located in different brain areas, including the hypothalamus, it is likely that their metabolic effects are mediated by central mechanisms (at least partially).

In summary, the research community has greatly advanced our understanding of how the gut–brain axis works, based on results from physiologically relevant aspects discovered more than 4 decades ago (203) to the newest results from chemically developed multi-agonists drugs. These advances now make it possible to envision a realistic treatment against the metabolic syndrome pandemic.

## Declaration of interest

The authors declare that there is no conflict of interest that could be perceived as prejudicing the impartiality of this review.

## Funding

This work was supported by grants from FEDER/Ministerio de Ciencia, Innovación y Universidades-Agencia Estatal de Investigación (RTI2018-099413-B-I00 and RED2018-102379-T), Xunta de Galicia http://dx.doi.org/10.13039/501100010801 (2016-PG057 and ED431C 2020/12), Fundación BBVA, Fundación Atresmedia, European Foundation for the Study of Diabetes http://dx.doi.org/10.13039/501100001648, Fundación La Caixa, the European Community’s H2020 Framework program under the following grant: ERC Synergy Grant-2019-WATCH- 810331. Centro de Investigación Biomédica en Red (CIBER) de Fisiopatología de la Obesidad y Nutrición (CIBERobn) is an initiative of the Instituto de Salud Carlos III http://dx.doi.org/10.13039/501100004587 (ISCIII) of Spain which is supported by FEDER funds.
